# Multiplexed Affinity Characterization of Protein Binders Directly from a Crude Cell Lysate by Covalent Capture on Suspension Bead Arrays

**DOI:** 10.1021/acs.analchem.0c03992

**Published:** 2021-01-05

**Authors:** Tuomas Huovinen, Laurens Lindenburg, Ralph Minter, Florian Hollfelder

**Affiliations:** † Department of Biochemistry, University of Cambridge, 80 Tennis Court Road, CB2 1GA Cambridge, U.K.; ‡ Antibody Discovery and Protein Engineering, R&D, AstraZeneca, Milstein Building, Granta Park, Cambridge CB21 6GH, U.K.

## Abstract

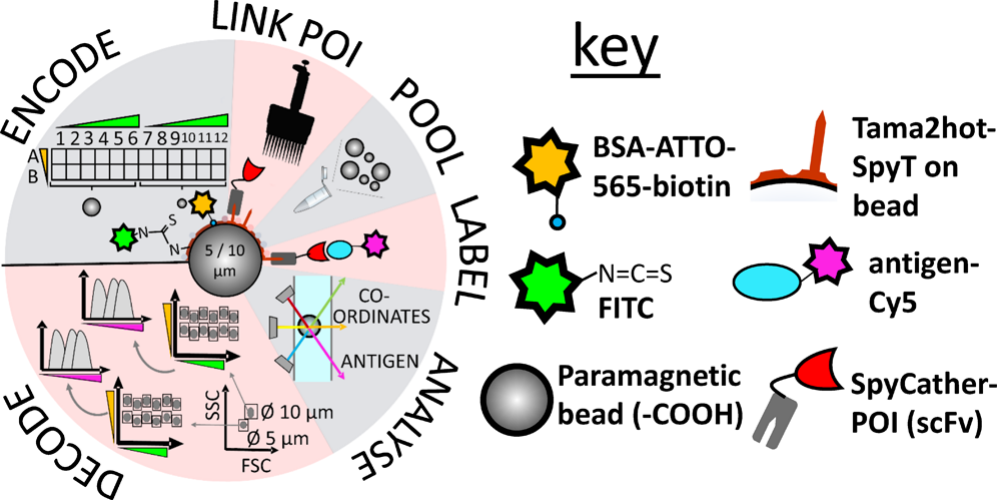

The precise determination of affinity and specificity is a crucial step in the development of new protein reagents for therapy and diagnostics. Paradoxically, the selection of protein binders, e.g., antibody fragments, from large combinatorial repertoires is a rapid process compared to the subsequent characterization of selected clones. Here we demonstrate the use of suspension bead arrays (SBA) in combination with flow cytometry to facilitate the post-selection analysis of binder affinities. The array is designed to capture the proteins of interest (POIs) covalently on the surface of superparamagnetic color-coded microbeads directly from expression cell lysate, based on SpyTag-SpyCatcher coupling by isopeptide bond formation. This concept was validated by analyzing the affinities of a typical phage display output, i.e., clones consisting of single-chain variable fragment antibodies (scFvs), as SpyCatcher fusions in 12- and 24-plex SBA formats using a standard three-laser flow cytometer. We demonstrate that the equilibrium dissociation constants (*K*_d_) obtained from multiplexed SBA assays correlate well with experiments performed on a larger scale, while the antigen consumption was reduced >100-fold compared to the conventional 96-well plate format. Protein screening and characterization by SBAs is a rapid and reagent-saving analytical format for combinatorial protein engineering to address specificity maturation and cross-reactivity profiling of antibodies.

Display technologies are powerful tools in protein drug discovery and have been widely applied to select antibodies and alternative scaffold proteins from synthetic libraries.^1,2^ Phage display antibody libraries with diversities approaching 10^11^ have been constructed for enriching the target binding clones by biopanning. Typically, after the initial selection by panning, additional labor-intensive screening of large numbers of individual library members is required to identify clones with the desired affinity and specificity.^3,4^ Paradoxically, even with a limited number of clones, the post-selection screening consumes more time and resources than the primary screening of repertoires of billions of members by panning selection. This post-selection screening is routinely performed in 96-well microtiter plates using enzyme-linked immunosorbent assay (ELISA), either directly with phage display clones or in a format with soluble protein for more precise characterization.^5^ Screening by phage ELISA requires 10–25 μg of protein in a 96- or 384-well microtiter plate.^6,7^ By contrast, only 500 ng of the target protein (coating a single microtiter well) is sufficient for one round of panning with 10^12^ antibody-displaying phages.^7^ Thus, approximately 20–50-fold more target protein is needed for the characterization of just one library member compared to the actual primary selection. For targets that are difficult-to-express or synthesize, such as G-protein-coupled receptors (GPCRs)^8^ and marine ciguatoxins^9^ the availability of the antigen may severely limit the extent of screening experiments. Miniaturization and multiplexing the primary screening effort would lead to considerable savings in time and material.

As a result of their high multiplexing capacity and low sample volume requirements, suspension bead arrays (SBA) have become a popular choice in medical research, e.g., for cytokine^10^ and allergen testing.^11^ SBAs typically consist of microbeads that are encoded with different ratios of organic fluorophores, creating a collection of unique spectral bead “addresses”.^12^ A test panel for multiple biomarkers is obtained by conjugating each batch of address beads with a different capture antibody. The beads are subsequently pooled for sample incubation, followed by the addition of target-specific detection antibodies. The detection antibodies, in turn, are coupled to a universal fluorophore (e.g., xMAP technology, Luminex)^13^ and, consequently, multiple analytes can be quantitated in a single flow cytometry experiment; the identity of the bead-bound analyte is determined by reading the fluorescence of the address bead, and the quantity of the analyte by reading the fluorescence of the detection antibody.

More recently, bead arrays have been applied to proteomics research for profiling plasma immunoglobulins against selected target proteins^14^ and for fluorescence-based Western blotting.^15^ Despite the clear advantages over 96-well plate ELISA screening, with respect to material savings and reduced manual liquid handling, SBAs have not been widely applied to recombinant binder screening due to the requirement of a proprietary analytical multiplexing instrument (e.g., Luminex). In a study by Ayriss et al., the binding specificity of recombinant single-chain variable fragment antibodies (scFvs) was analyzed in an 8-plex Luminex bead assay with bead-bound target antigens using myc-tag for detecting the antigen-bound scFvs.^16^ In this arrangement, each clone is individually examined against the eight targets. However, multiplexing binders instead of the antigens would reduce the number of screening samples, and the amount of antigen needed even further.

In this work, SBAs are explored as a flexible tool for miniaturizing the primary screening of antibody fragments in a multiplexed, flow cytometry-based analysis format using paramagnetic microbeads. We designed a novel SBA set-up that obviates the need to purify the binders prior to bead conjugation. To this end, we take advantage of the isopeptide bond-forming SpyTag-SpyCatcher protein pair,^17^ making the purification of proteins for covalent conjugation on sets of beads (as in the commercial SBAs) superfluous. First, altogether 48 bead populations with unique optical signatures (referred to as the address bead set) were created. The optical addresses of unique bead populations provided a readout of the origin of the displayed protein (e.g., for tracking the source microwell by flow cytometry) based on the bead size and the ratio of the two conjugated fluorophores on the bead. When these beads are decorated covalently with the proteins of interest (POIs) via SpyCatcher/SpyTag pairs,^17^ binding titrations can be carried out with a standard analytical three-laser flow cytometer that simultaneously provides quantitative insight into the function (e.g., binding of a fluorescently labeled target molecule) and the source location of the binder in question.

## Experimental Section

### Coating Beads with Tamavidin-2-HOT-SpyTag

Carboxylate-modified microbeads from three providers with different sizes were tested for multiplexing: (i) superparamagnetic polystyrene particles (mean Ø 1.43, 5.18, and 10.31 μm) from Microparticles GmbH (Berlin, Germany), (ii) paramagnetic beads (mean Ø 7.4 and 18.8 μm) from Spherotech (Lake Forest, IL), and (iii) superparamagnetic Dynabeads M-270 (Ø 2.8 μm) from Thermo Fisher Scientific (Waltham, MA). The beads with 5.18 and 10.31 μm diameter were further used for bead coating (henceforth referred to as the Ø 5 and 10 μm beads, respectively). To prepare the beads for covalent coupling with tamavidin-2-HOT-SpyTag (T2H-SpyTag), the beads (24 mg) were washed with 4 × 1 mL of water containing Tween-20 (0.01% w/v), and the carboxyl groups were activated to react with primary amines by resuspending the bead pellet in 0.5 mL of water containing N-(3-dimethylaminopropyl)-N′-ethylcarbodiimide hydrochloride (EDC) (100 mg; Sigma) and sulfo-NHS (21 mg; Sigma). The beads were incubated for 20 min at RT (23 °C) with rotation, interrupted by frequent vortexing, and washed once with 1 mL of water. The beads were mixed with T2H-SpyTag (2.5 mg in 1 mL in 50 mM sodium phosphate buffer, pH 5.7), vortexed, and incubated overnight at RT with rotation. The reaction was quenched by incubating the beads in Tris-Cl (1 mL of a 0.5 M solution, pH 8) for 10 min with rotation. The beads were washed with 3 × 1 mL of PBST^0.1^ (phosphate-buffered saline (PBS) + 0.1% w/v Tween-20) and resuspended in PBST^0.05^ (1 mL of PBS + 0.05% w/v Tween-20).

### Labeling Beads with Fluorophores

T2H-SpyTag-coated beads were divided into 3 mg batches and resuspended in carbonate buffer (950 μL of a 0.1 M buffer, pH 9.4). Fluorescein-5-isothiocyanate (FITC) powder (Sigma-Aldrich, St. Louis, MO) was freshly dissolved in dimethyl sulfoxide (DMSO), and the stock solution was further diluted in DMSO to create a series of master solutions (20× concentrates). The master solutions were mixed with 950 μL of bead batches and immediately vortexed, resulting in the final FITC concentrations of 28, 3.2, 0.8, 0.4, and 0.02 μg/mL. The beads were protected from light and incubated overnight at RT with rotation. The reaction was quenched by incubating the beads in Tris-Cl (1 mL, 0.5 M, pH 8) for 10 min with rotation. The beads were washed twice with 1 mL of PBST^0.1^ and resuspended in 1 mL of PBST^0.05^. The observed dynamic labeling range for FITC in 1 mL of carbonate buffer at pH 9 (Ø 5 μm beads, incubated for >12 h at RT with rotation) ranged from 0.02 μg of FITC/3 mg of beads to 28 μg of FITC/3 mg of beads.

To add the second fluorophore gradient on the FITC conjugated beads, the bead suspensions were split into 100 μL aliquots (∼300 μg of beads per aliquot), pelleted, and resuspended in 50 μL of PBST^0.05^ containing different concentrations of bio-BSA-ATTO-565. The synthesis of bio-BSA-ATTO-565 is described in the SI. To facilitate bead gating in the analysis phase, bio-BSA-ATTO-565 was added at different concentrations on every other FITC-bead batch according to increasing FITC intensity. The first series of beads was labeled with 0, 5, 40, and 200 nM bio-BSA-ATTO-565 and the second with 1, 10, 80, and 400 nM bio-BSA-ATTO-565. For labeling Ø 10 μm beads, the first series of beads was labeled with 0.1, 1, 4, and 20 nM bio-BSA-ATTO-565 and the second with 0.5, 2, 8, and 40 nM bio-BSA-ATTO-565, respectively. The labeling reactions were protected from light and incubated for 15 min at RT with rotation. The beads were washed with 3 × 1 mL of PBST^0.1^ and resuspended in 200 μL of PBST^0.05^. The dynamic labeling range for bio-ATTO-565 in 50 μL of PBST^0.05^ (Ø 5 μm beads, incubated for 15 min at RT with rotation) was from 0.05 pmol of bio-ATTO-565/300 μg of beads to 20 pmol of bio-ATTO-565/300 μg of beads.

### Multiplexed Bead Screening

An anti-digoxigenin (anti-DIG) scFv library was constructed on the parental clone 198C9 using NNS–NNS site-saturation mutagenesis, and two rounds of phage display selections were performed to obtain affinity-improved variants (see the SI for library design and selection). After phage display selection, the scFv genes were extracted from the phage input and output libraries (display was performed with phagemid pHB32*x*) and cloned via SfiI sites into the pHBSC screening vector to create scFv-SpyCatcher fusions. The screening repertoires were transformed into α-Select Silver *Escherichia coli* strain (Bioline) for small-scale expression in 96-well plates. Colonies were picked, proteins expressed, and cells lysed with a combination of lysozyme/benzonase (for treatment and freeze–thawing see ref (18), except that deep well plates, 400 μL volume per well, were used instead of standard microtiter plates). The cell debris was pelleted at 3200 *g* for 30 min, and 250 μL of supernatants were transferred to fresh polymerase chain reaction (PCR) plates (Thermo-Fast 96, Semi-Skirted PCR Plate, Thermo Fisher Scientific).

The scFv-SpyCatcher fusion proteins thus expressed were attached to T2H-SpyTag beads by mixing 50 μL of the lysate supernatant with 5 × 10^4^ fluorophore-labeled address beads per well on a PCR plate. The reaction mixture was incubated for 30 min at RT with shaking. The beads were collected using a Dynamag 96-well side magnet (Thermo Fisher Scientific), washed once with 200 μL of PBST^0.1^, and resuspended in 50 μL PBST^0.05^. The beads were pooled into one microcentrifuge tube by combining samples from one row of a 96-well plate for 12-plex assay (input and output A) and two rows for 24-plex assay (output B), respectively. The supernatant was removed from the bead collection tube and the bead pellet resuspended in 1 mL of 5 nM antigen solution, DIG-dsDNA-Cy5, in PBST^0.05^. The mixture was incubated for 1 h at RT with rotation, washed twice with 1 mL of PBST^0.1^ and resuspended in 200 μL of PBST^0.05^ for flow cytometry on a BD FACScan (BD Biosciences, San Jose, CA).

### Saturation Binding Analysis

scFv-SpyCatcher fusions (100 μL) expressed in α-Select Silver *E. coli* cells were released into cell supernatant as described above and mixed with 2 × 10^5^ T2H-SpyTag beads for bead conjugation. Following the incubation and washing steps as described above, the beads, coated with different scFv-SpyCatcher variants, were pooled and split into equal aliquots for adding the DIG-dsDNA-Cy5 antigen dilutions provided in 200 μL or 2 mL volumes in PBST^0.05^. The antigen dilution series was incubated with the beads at least for 2 h at RT with rotation. The beads incubated with different antigen concentrations were processed one by one for flow cytometry. The beads were collected using a magnet, and the supernatant was removed. The beads were resuspended in 200 μL of PBST^0.05^ and immediately analyzed with a FACScan Cytek. The median fluorescence intensity (MFI) values for each gated bead population at each antigen concentration was obtained with FlowJo10 software, exported to GraphPad Prism6 (GraphPad software), and the data were fitted to eq 1, assuming one site-specific binding
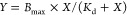
1where *Y* is the fluorescence, *B*_max_ is the maximum specific binding in the same units as *Y*, *X* is the antigen concentration, and *K*_d_ is the equilibrium dissociation constant. The fluorescence signal arising from the nonspecific binding of the labeled antigen to the beads was obtained from intra-assay control beads that were not coated with scFv-SpyCatcher. This nonspecific signal was subtracted from the total signal obtained from scFv-SpyCatcher beads prior to fitting to the one site-specific binding equation.

## Results and Discussion

### Multifunctional Beads for Optical Decoding of Clonal Identity and Binding Function

**Figure 1:**
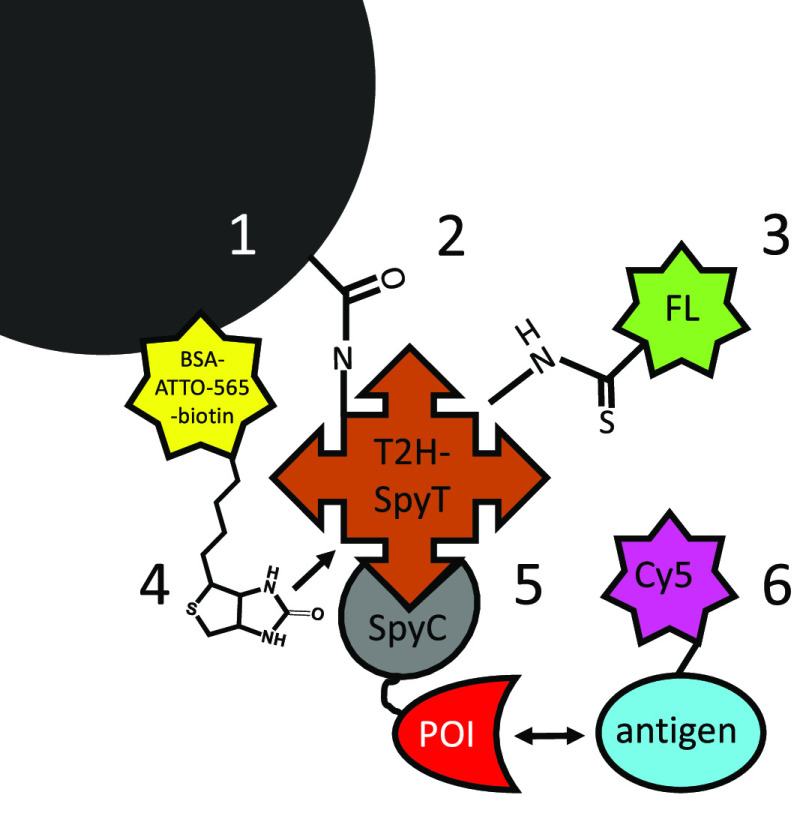
Assembly and functionalization of beads for the preparation of spectral addresses, POI addition, and labeled antigen detection. (1) Two differently sized beads (Ø 5 and 10 μm) allowed forward and side scatter-based discrimination in the flow cytometer. (2) The EDC cross-linker resulted in the formation of a carboxamide linkage between carboxylic acids on the bead and primary amine on Tamavidin-SpyTag. (3) Beads were functionalized with fluorescein through a covalent thiourea linkage with remaining free primary amines on Tamavidin-Spytag and FITC. (4) Beads were functionalized with ATTO-565 through noncovalent binding of the ATTO-565-BSA-biotin conjugate to the biotin-binding sites of tamavidin. (5) The protein binder of interest (POI) was introduced through the spontaneous formation of an isopeptide bond between SpyCatcher (fused to the POI) and SpyTag fused to Tamavidin. (6) Binding of the antigen to the POI could be monitored through the Cy5-dye linked to the antigen.

Figure 1 illustrates the construct that forms the basis of our screening platform. Its centerpiece are paramagnetic microbeads that allow further surface modification (see below), and are straightforwardly handled with a bar magnet (and amenable to automation).^19^ Our objective was to enable specific covalent attachment of the recombinant proteins directly from the cell lysate to the beads, which was achieved by implementing spontaneous covalent coupling via SpyTag-SpyCatcher technology, which has been shown to perform robustly under different buffer conditions.^17^ To this end, the SpyTag was produced as a C-terminal protein fused to tamavidin-2-HOT, a heat-stabilized protein tetramer binding biotin with high-affinity,^20,21^ and containing seven surface-exposed lysine residues in each subunit, which provide anchors for efficient chemical conjugation to carboxyl-modified beads via carbodiimide-mediated amide bond formation. Consequently, recombinant proteins of interest (POI) expressed as POI-SpyCatcher fusions in *E. coli* were covalently attached to microbeads coated with the SpyTag-fused carrier protein directly in the expression lysate. Optical address signatures for tracking the source locations of the clones were created on the beads with combinations of two fluorophores at discernible intensities. The first fluorophore gradient was attached via EDC cross-linking chemistry to target the remaining surface-exposed amine groups of tamavidin on the bead, and the biotin-binding site of tamavidin-2-HOT was taken as the second orthogonal attachment site to include the second spectral coding dimension.

**Figure 2:**
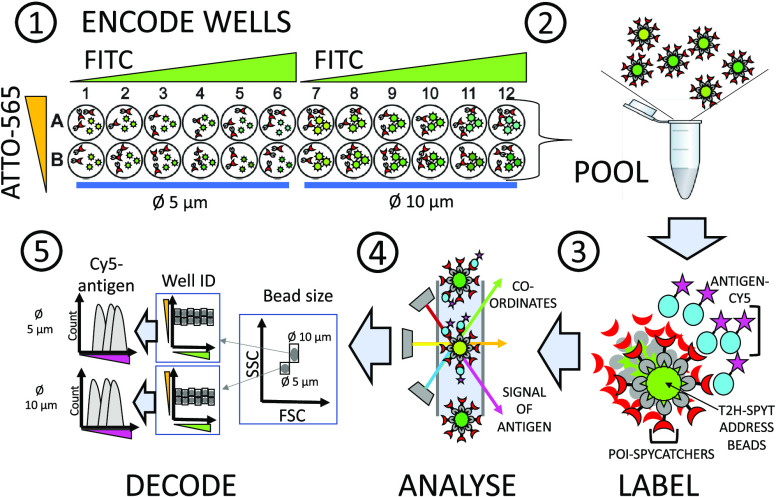
Schematic workflow for screening POI-SpyCatcher clones with a 24-plex spectrally encoded SBA functionalized with SpyTag. (1) Encoding expression wells with address beads. POI-SpyCatcher fusions were expressed in *E. coli* cells in 96-well microtiter plates. After cell lysis and pelleting the debris, the fusion proteins in the soluble fraction were directly captured on the beads. Each well containing the expression supernatant was supplemented with 10^4^ address beads labeled with different quantities of fluorescent dyes, FITC and bio-ATTO-565, coding for the column and row identity, respectively. Consequently, the source well of the probed bead-bound POIs could be later traced by flow cytometry. (2, 3) Pooling and labeling. After POI-SpyCatcher fusion proteins were stably captured on the bead surface by the formation of isopeptide bonds, the beads were washed to remove the unbound POI-SpyCatchers and pooled for labeling with the Cy5 antigen. (4) Flow cytometry analysis. Readout via two laser lines (blue, λ = 488 nm for detecting FITC; yellow, λ = 563 nm, for detecting ATTO-565) decoded the source well coordinates of the examined bead, and a third laser (red, λ = 633 nm) detected the signal indicative of antigen-binding (Cy5). (5) Gating and decoding. The recorded events were first gated by the bead size (in an FSC/SSC view) and then by the FITC/ATTO-565 – fluorescence ratio giving the well coordinates. The extent of antigen-binding for each POI-SpyCatcher was determined by analyzing the median fluorescence intensity (MFI) of each gated bead population in the Cy5 channel (as shown, right). The wells supplemented with Ø 5 and 10 μm beads were separately analyzed for antigen-binding as the MFI levels are not comparable on different sized particles.

Our model scFv anti-DIG 180B1 used for studying the SBA concept expressed better in the periplasm of *E. coli* as the larger scFv-SpyCatcher (38.2 kDa) fusion protein than as scFv-SpyTag (30.2 kDa) fusion protein (Figure S1), which supported the use of SpyTag as the anchor point on the microbead. Two spectrally distinguishable dyes, the yellow laser excitable biotinylated ATTO-565 (maximum excitation/emission, λ: 564/590 nm) and the blue laser excitable fluorescein isothiocyanate (FITC, 490/525 nm) were used to create the address bead array. In this arrangement, Cy5 (649/666 nm, excitable by a red laser) was reserved for the detection of successful antigen-binding function. Schematic workflow for SBA screening is illustrated in Figure 2. It was further validated that after fusion of SpyTag to tamavidin-2-HOT the biotin-binding function of tamavidin was retained (Figure S2A) and that anti-DIG ScFv-SpyCatcher was stably bound on the SpyTag beads, as detected by the fluorescence of the labeled antigen, DIG-dsDNA-Cy5, on the bead surface (Figure S2B).

### Color and Bead Size Encoding to Create Spectral Address Beads

The ability of the bead size to provide an additional dimension for multiplexing was explored. Six stocks of carboxyl-activated paramagnetic beads (Ø 1.43–18.8 μm) were analyzed by flow cytometry (FACSCAN Cytek). Focused spots for unambiguous gating were observed with bead diameters between 3 and 18.8 μm (Figure S3A), while the smallest bead size of Ø 1.43 μm did not form a focused spot on the scatter plot but rather overlapped with other bead populations (Figure S3B). As Ø 7.4 μm beads overlapped with Ø 10 μm bead sizes and the bead count of the Ø 19 μm stock was at least 5-fold lower than that of the other stocks. Only Ø 5 and 10 μm beads were employed in subsequent experiments described below.

**Figure 3:**
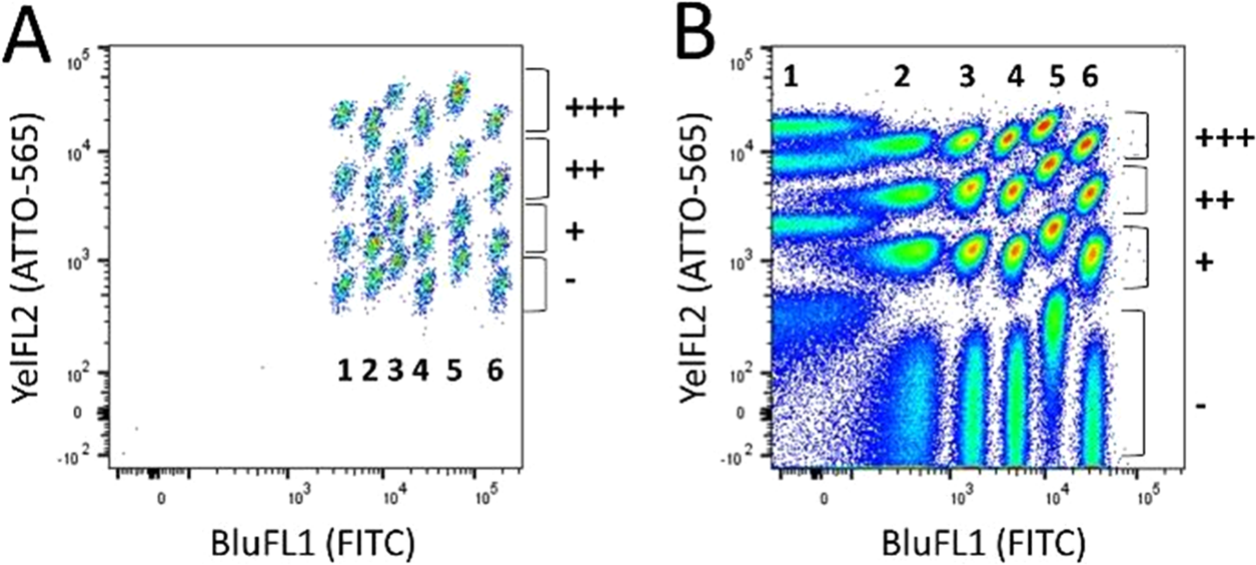
Dot plot of the spectral addresses of 24-plex bead arrays coated with T2H-SpyTag and labeled with four bio-ATTO-565 (+++, ++, +, −) and six FITC (1–6) intensities for color-coding the beads. (A) Ø 10 μm bead array. (B) Ø 5 μm bead array. The unique combination of FITC/ATTO-565 – intensities indicates the array positions of the clones on the source microwell plate, e.g., on a 96-well plate corresponding to the well coordinates from A1 (1+++) to D6 (6−).

Six distinct FITC intensities and four distinct ATTO-565 intensities were achieved on Ø 5 and 10 μm bead populations creating 2 × 24-plex bead arrays (Figure 3). In general, 3-fold differences in FITC concentrations were visually distinguishable on the BluFL1/YeFL1 scatter plot. For bio-ATTO-565 labeling, 5–10-fold dilutions were used to prepare the distinct populations. The pooled Ø 5 and 10 μm beads could be first gated on the FSC/SSC scatter plot to separate size groups and then gated further on the BluFL1/YeFL1 scatter plot for single-well resolution.

### Validation of SBA for Recombinant Binder Screening

**Figure 4:**
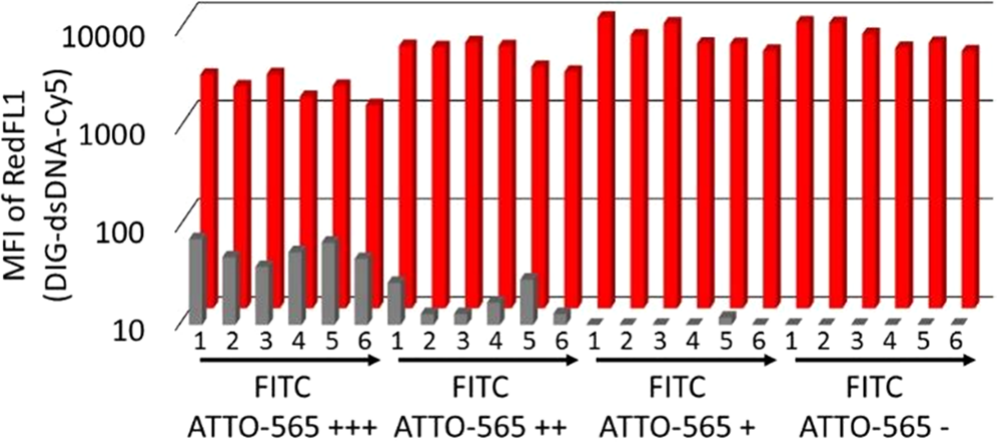
Analysis of antigen-binding signals obtained from the 24-plexed bead array (Ø 5 μm) with spectral addresses. The beads were precoated with the anti-DIG scFv-SpyCatcher protein and incubated with 50 nM DIG-dsDNA-Cy5 antigen. The medium fluorescence intensity (MFI) of the RedFL1 channel detecting Cy5 fluorescence is shown with the red histogram for each gated bead population. The same assay was carried out with equivalent uncoated bead populations (the nonspecific DIG-dsDNA-Cy5 signal), which is shown in gray, respectively. The spectral populations (1–6 for FITC and +++, ++, +, – for ATTO-565) are identical to the ones described in the legend to Figure 3.

Next, we analyzed whether the implemented surface-labeling strategy for constructing the address bead repertoires had an effect on the antigen-binding signal. To this end, 50 000 beads (Ø 5 μm) of each subpopulation were coated with anti-DIG scFv-SpyCatcher and labeled with DIG-dsDNA-Cy5. Flow cytometry analysis revealed that functional signals were obtained from all bead populations (Figure 4). However, there was a systematic 2-fold difference in the antigen-binding signal between the beads containing the highest FITC-labeling degree (28 μg of FITC per 3 mg of beads) and the lowest labeling degree (Figure 4, FITC 1 vs 6). Similarly, a 3-fold decrease in the antigen-binding signal was observed between the non-ATTO-565-labeled beads (ATTO-565 −) and the highest degree of labeling (20 pmol bio-BSA-ATTO-565 per 300 μg of beads; ATTO-565 +++). The reason for the observed difference in the maximum antigen-binding capacity is most likely due to the high density of bead-bound fluorophores, causing steric hindrance of scFv-SpyCatcher binding to the beads. Alternatively, in the highly labeled sample FITC may have attached to the lysines close to the central SpyTag sequence AHIVMVDAYKPTK, of which especially Lys10 is interacting with the SpyCatcher,^17^ resulting in lower scFv-SpyCatcher coupling efficiency.

The Cy5 signal was also measured of beads that were not coated with scFv-SpyCatcher, yet incubated, with the Cy5 antigen. This background signal on the RedFL1 channel was the highest on the brightest ATTO-565 beads (60 ± 18 median RedFL1 au) compared to the non-labeled beads (4.9 ± 1.3 median RedFL1 au). The variation in the background signal among the address beads was, however, considered insignificant for primary screening of clones as at least 240-fold signal-to-background ratios were obtained with all bead populations. The increased background fluorescence at the highest ATTO-565 intensity is speculated to be caused by nonspecific binding of the DIG-dsDNA-Cy5 to the fluorophore beads, as there is virtually no spectral overlap between the selected fluorophores and the chosen detection channels. We speculate that a more uniform functional signal could be attained by internally color-coded microbeads.

**Figure 5:**
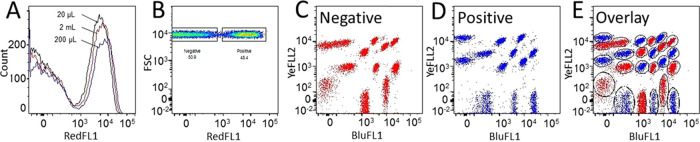
Confirmation of lack of cross-contamination of bead-bound scFv-SpyCatchers by gating positive and negative SBA populations from pooled samples. (A) Histograms of a 24-plex bead array (Ø 5 μm), in which every other bead population was coated with scFv-SpyCatcher (200 ng) and labeled after pooling with 25 nM DIG-dsDNA-Cy5 in 20 μL, 200 μL, and 2 mL reaction volumes, showing similar DIG-dsDNA-Cy5 (RedFL1) binding profiles. (B) The bead sample labeled with 200 μL antigen was gated in negative (C) and positive (D) bead populations based on the fluorescence in RedFL1 channel. (E) The overlay of the antigen-positive and negative gates in a dot plot with BluFL1 (FITC)/YelFL2 (ATTO-565) axes indicates stable covalent linking of scFv-SpyCatchers to the beads as all 24 bead populations can be unequivocally assigned to their respective wells of origin.

In the assembly construct (Figure 1), a stable covalent link between SpyCatcher-fused binder and SpyTag-functionalized bead is a key prerequisite for the screening of binders with pooled bead arrays. To confirm its stability and rule out SpyCatcher-scFv cross-contamination across beads, address bead arrays were applied to a 96-well microtiter plate, and every other well was incubated with anti-DIG scFv-SpyCatcher before pooling the beads. Subsequently, the beads were labeled with the DIG-dsDNA-Cy5 antigen and analyzed by flow cytometry. Two distinct peaks were observed in the RedFL1 histogram (Figure 5A), reflecting the two populations. These peaks were gated based on RedFL1 fluorescence intensity (Figure 5B) and superimposed back onto the dot plot with BluFL1/YelFL2 axes. The superimposition confirms the correct spectral address bead groups, i.e., source wells (Figure 5C–E): beads coated with scFv-SpyCatcher protein are highlighted in the positive gate (Figure 5D,E) and are clearly distinct from the beads derived from the negative gate (Figure 5C,E). This observation allows the conclusion that scFv-SpyCatchers were firmly bound to the SpyTag-coated beads and that the unambiguous identification is possible based on a dot plot with BluFL1/YelFL2 axes corresponding to their source wells. Remarkably, a 25 μL volume of 25 nM DIG-dsDNA-Cy5 (0.83 μL per spectral bead batch) antigen solution provided an almost identical profile to a 2 mL volume one (83 μL per spectral bead batch) (Figure 5A). This observation suggests that at the provided antigen concentration, the labeling reaction can be operated at a 100-fold reduced scale without sacrificing the functional signal.

### Multiplexed Screening and Characterization of Anti-Digoxigenin scFv Clones with SBAs

The suitability of this suspension bead array technology for multiplexed analysis of recombinant binders was demonstrated by screening anti-DIG scFv repertoires for affinity-improved binders. A site-saturation library of scFv clone 198C9 was constructed and enriched by two rounds of phage display against biotin-dsDNA-digoxigenin (see the SI for details of parental clone origin, phage display library construction, and phage display cycles). We were especially interested in obtaining affinity-improved binders recognizing digoxigenin as a DNA conjugate, which could be used as a general detection tool for DIG-labeled probes. The phage display selection was performed under two different conditions: either at 20 pM (output A) or at 1 pM (output B) antigen concentration in solution followed by capture on paramagnetic streptavidin beads. After selection, the enriched scFv clones and controls were cloned into the screening vector pHBSC creating scFv-SpyCatcher fusion proteins for periplasmic expression in 96-well plates (input, output A, and output B with 84 library clones each) and SBA screening (see Figure S4 for workflow).

**Figure 6:**
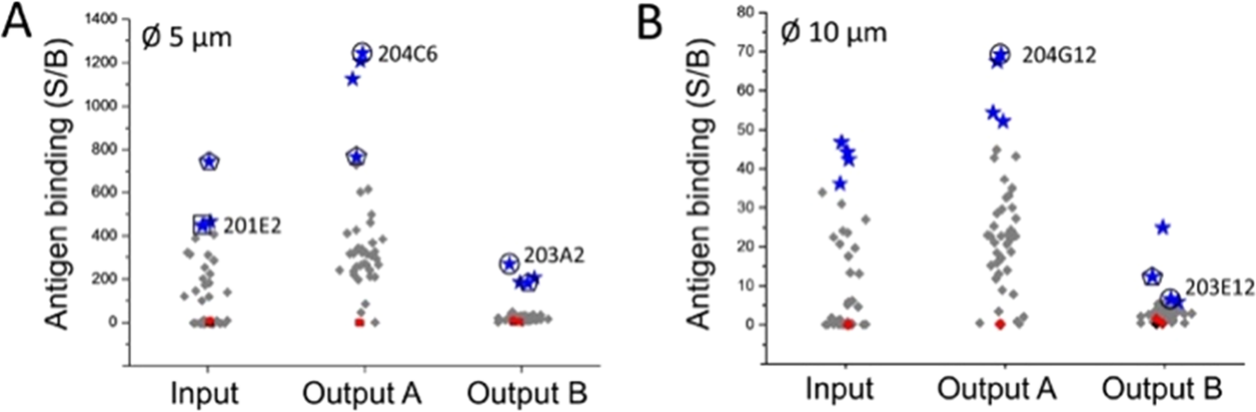
Primary screening of scFvs as SpyCatcher fusion proteins on SBAs after two rounds of phage display. The screening results were obtained with SBAs using beads with diameters of Ø 5 μm (panel A) and Ø 10 μm (panel B) using 5 nM digoxigenin-dsDNA-Cy5 as the label. The binders were screened from the phage display input library and two output libraries, which were obtained by allowing the scFv-phages to bind to the antigen, biotin-dsDNA–DIG, at 20 pM concentration (output A) or at 1 pM (output B) concentration in solution before capture. The clones labeled with blue stars (four top clones from both Ø 5 and Ø 10 μm bead sets) were analyzed in a secondary affinity validation assay. The red diamonds represent empty beads. The signal-to-background (S/B) is the MFI of each bead in RedFL1 divided by the mean MFI of empty beads. The circled clones were later identified as the highest affinity output clones with unique genotypes in the subsequent secondary assay. Clones marked with a pentagon failed to provide adequate dose-response for fitting to the binding equation (see Table S4). The saturation binding histograms recorded for clone 201E2 (squared) and 204C6 (circled) in the secondary assay are highlighted in Figure 7.

The primary SBA screening data of the three expression plates are shown in Figure 6, and an example of 24-plex sample analysis in Figure S5 (bead multiplexing and screening details are given in Table S3). The error caused by address bead labeling to the antigen-binding signal was minimized (to ≤2-fold) using a limited set of color-coded beads (two sizes [5 vs 10 μm], six FITC intensities, and two ATTO-565 intensities), leaving the highest fluorescent ATTO-565 bead sets out. Furthermore, stringent gating on the highest density of address bead populations (see the representation of BLuFL1/YelFL2, Figure S5) effectively mitigates the possibility of including members of the neighboring bead population in the antigen-binding analysis. The observation that the frequency of binders increases as a result of the phage display selection (Table S3) suggests that SBA is a suitable platform for discriminating target binding clones from nonbinders. However, a precise ranking of the clones by affinity requires a more quantitative analysis at different antigen concentrations because the signal level in single-point screening is dependent both on the expression level of individual clones and the manual adjustment of the fluorescence intensities in a flow cytometer prior to analysis. Therefore, the eight most positive clones from the primary screening of the input and output repertoires were selected for a secondary screening assay in which the developed SBA platform was applied for obtaining complete antigen response curves. These ligand (antigen) saturation binding experiments, in which the extent of the binding reaction was determined as a function of the concentration of the ligand (antigen), were also used for the determination of binding dissociation constants at equilibrium (*K*_d_).

**Figure 7:**
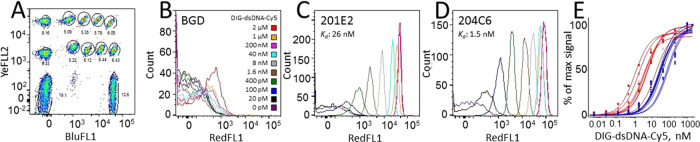
Secondary screening of anti-DIG ScFv-SpyCatcher hits with multiplexed saturation binding analysis. (A) Gates for 12 bead populations (eight clones and controls) in an equilibrium binding experiment performed with DIG-dsDNA-Cy5 at 2 μM–4 pM concentrations. Each address bead batch was separately coated with an scFv-SpyCatcher clone expressed in the cell lysate, pooled together, and split again into ten aliquots for labeling with different antigen concentrations. The proportions of bead batches (%) of all events are indicated below the gates. (B–D) Saturation binding histograms of individual clones. Each curve corresponds to one antigen concentration. (B) Nonspecific background signal (BGD) of the label (equivalent to a nonbinding clone), (C) scFv-SpyCatcher clone 201E2 (*K*_d_ for DIG-dsDNA-Cy5: 26 nM), and (D) scFv-SpyCatcher clone 204C6 (*K*_d_ for DIG-dsDNA-Cy5: 1.5 nM). (E) Specific binding curves obtained by two 12-plex assays for the eight highest-ranking scFv-SpyCatcher clones chosen by the primary screening of the phage display input (blue) and output A (red) libraries. Only six of eight curves are shown for clarity. The specific saturation binding curves were obtained for each clone by plotting the MFI of the RedFL1 channel obtained from each gated bead population over a range of antigen concentrations. The nonspecific binding signal was subtracted from the specific signal prior to fitting.

The antigen saturation binding assay was performed in a 12-plex format (8 clones, two empty beads, and two controls) by capturing the selected scFv-SpyCatcher clones from the lysate on the address beads, pooling the clones together, and splitting them again in ten aliquots for antigen labeling. The aliquots were supplemented with increasing concentrations of DIG-dsDNA-Cy5 and incubated for 2 h to allow the binding reaction to reach equilibrium, followed by flow cytometry analysis. In the decoding step, the bead populations were gated based on their BluFL1/YelFL2 fluorescence ratio (Figure 7A), allowing their antigen-binding response (RedFL1 signal) to be individually analyzed. An exemplary panel of histograms obtained at different antigen concentrations for two anti-DIG scFv clones and beads not displaying scFv is shown in Figure 7 (BCD). The successful enrichment of binders in the phage display experiment was confirmed by ranking the saturation binding curves of the top scFv-SpyCatcher clones (Figure 7E). Post-selection clones (6/8) from output A and 5/8 clones from output B had at least 2-fold higher affinity for DIG-dsDNA-Cy5 than the top clones analyzed from the unselected repertoire (Table S4). It was also observed that the primary screening results were adequate to distinguish binders from nonbinding clones but displayed only a rough correlation with antigen-binding affinities that were later obtained by biophysical analysis. For example, the antigen-binding signals (MFI RedFL1) obtained in the primary output B (Figure 6) were overall lower than the signals obtained in the input and output A screening (performed on a different day). However, binders with equal affinity for DIG-dsDNA-Cy5 were identified among both outputs in the ligand saturation binding experiments (Table 1).

**Table 1:** Affinity Characterization of Selected scFv-SpyCatcher Clones for Binding to Digoxigenin^a^

ScFv	mutations	on-bead *K*_d_^b^ (200 μL), nM	on-bead *K*_d_^c^ (2 mL), nM	*K*_d_ gain^d^	BLI *K*_d_^e^ nM
180B1	+A53V_VH_+T55S_VH_+T57N_VH_	18.2 ± 2.9	11.2/14.9 (13.1)	N.D.	N.D.
198C9	+E1K_VH_	16.1 ± 2.8	12.4/13.1 (12.7)	parent	21.4 ± 9.8
203E12	+E1K_VH_+**A114P_VL_**	4.0 ± 1.6	2.7/1.5 (2.1)	6.0	N.D.
204C6	+E1K_VH_+**A114P_VL_**+N106S_VH_	3.0 ± 0.4	1.5/1.4 (1.4)	9.1	N.D.
204G12	+E1K_VH_+**A114P_VL_**+A53V_VH_	3.6 ± 0.2	2.7/1.7 (2.2)	5.8	N.D.
203A2	+E1K_VH_+**A114P_VL_**+A53V_VH_+N106S_VH_	4.9 ± 0.4	1.6/1.2 (1.4)	9.1	1.2 ± 0.6

a The on-bead *K*_d_ was separately determined from antigen (DIG-dsDNA-Cy5) saturation binding assays measured in equilibrium by performing the antigen labeling reactions in total reaction volumes of 2 mL and 200 μL.

b The mean and standard deviation of three independent experiments.

c *K*_d_ values obtained in two independent experiments (I/II) and their mean.

d The *K*_d_ gain was calculated as the ratio of the parent 198C9 to the variant *K*_d_ (derived from the mean of on-bead experiments performed in a 2 mL reaction volume).

e Biolayer interferometry experiments were carried out with the Octet platform (ForteBio) by immobilizing biotin-dsDNA–DIG on streptavidin tips and using scFv-SpyCatcher constructs as analytes at five different concentrations to record binding curves.

The robustness of the color-coded bead arrays for saturation plot analysis and *K*_d_ determination was studied in repeated experiments by coupling the binders from the lysate each time on spectrally different address bead populations. Similar response curves and *K*_d_ values were obtained for each scFv-SpyCatcher clone tested, irrespective of the particular address bead population used for coupling (Figure S6). The equilibrium binding assay was also studied by providing a DIG-dsDNA-Cy5 gradient in 200 μL and 2 mL volumes to assess possible antigen depletion effects. Incubating the suspension bead array in 2 mL volumes resulted in higher affinity values for the parental clones 180B1 and 198C9 (*K*_d_ ∼ 13–18 nM, 1.3–1.4-fold higher affinity in 2 mL than that in 200 μL) and the affinity-improved clones (*K*_d_ ∼ 1.4–5 nM, 1.6–3.5-fold higher affinity). This indicates that maximizing the volume of the provided antigen dilution helps to minimize the antigen depletion effects. Depletion is a known factor to be taken into account for saturation binding experiments,^22^ especially if the dissociation constants to be analyzed are in low nanomolar or picomolar ranges. In a typical primary hit screening assay, the antigen depletion effect is not a significant factor for assay interpretation (see Figure 6), as very few beads carry high-affinity binders and the data are qualitative rather than quantitative in nature.

Over 5-fold improvement in the digoxigenin binding affinity was observed with the output clones 203A2, 204C6, 203E12, and 204G12 (when compared to the parental clone 198C9 by saturation binding analysis, Table 1). Sequence analysis of the selected scFvs revealed that affinity improvement was correlated with the presence of an A114P_VL_ mutation in the CDR-L3 loop (Table 1). The validity of the *K*_d_ values obtained with the multiplexed bead assay was confirmed using biolayer interferometry (BLI) and kinetic binding analysis (Figure S7) of a parental clone 198C9 (*K*_d_: 21.4 ± 9.8 nM) and affinity-improved clone 203A2 (1.2 ± 0.6 nM), and found to be in good agreement with the saturation binding experiments.

## Conclusions

This work demonstrates the use of flow cytometry for obtaining specificity data with SBAs by capturing POI-SpyCatcher fusions directly from cell lysate on the surface of superparamagnetic color-coded beads via SpyTag/SpyCatcher chemistry. To our knowledge, this is the first study to demonstrate the robust covalent linking of recombinant proteins in complex media on-bead arrays, which is an indispensable requirement for the application of SBAs for multiplexed binder screening, as by this means only the cross-contamination of POIs across beads can be avoided after pooling. This concept could be further adjusted to commercially available internally labeled address bead custom arrays (e.g., Luminex beads)^23^ by functionalizing the beads with T2H-SpyTag. Compared to conventional microplate screening, SBA screening has low antigen consumption; for example, a 96-well expression plate of single scFv-SpyCatcher clones was analyzed in a 24-plex format by pooling the beads into four 1 mL antigen-binding reactions, which could be further reduced to a 20 μL reaction scale (0.83 μL clone) without significant loss in performance (Figure 5A).

Suspension bead technology was applied to provide data for the determination of binding dissociation constants (*K*_d_) at equilibrium by ligand saturation binding experiments. In this multiplexed SBA format, the ligand binding is simultaneously examined for all clones of interest in single series of antigen dilutions minimizing experimental errors. Furthermore, differences in the maximum functional signal across the bead array (due to the surface-labeling strategy) do not play a role in saturation binding analysis as the antigen-binding signals for each clone are recorded on the same spectral address bead batch at different antigen concentrations. Most importantly, in flow cytometry, the fraction of the bound fluorescent analyte at equilibrium is measured directly on the bead surface. Therefore, washing steps (that may disturb the equilibrium) can be minimized or altogether omitted. In contrast, in ELISA or other methods requiring solid-phase separations, the antigen-binding equilibrium may be severely disturbed by numerous washing steps depending on the dissociation rate of interaction.^24,25^

Previous miniaturization efforts of screening formats had mainly focused on microarrays.^26−28^ However, microarray technology requires specialized printing and reading instruments with substantial costs for capital investment. In contrast, this SBA format is compatible with any standard three-laser flow cytometer, simplifying the practicalities of analysis. In addition, the construction of bead arrays from readily available components provides the researcher with ample degrees of freedom to choose customized combinations of fluorescent labels for the bead coordinates and functional signal, to fit specific assay requirements. When additional laser lines are available in modern flow cytometers, the SBA platform can be multiplexed further, allowing the simultaneous detection of, e.g., expression levels or cross-reactivities to other antigens, in parallel to antigen-binding.

This straightforward and versatile technology can thus be implemented straightforwardly in non-specialist laboratories to generate and characterize affinity reagents with tailor-made specificity. In-house access to affinity reagents, bypassing commercial providers, and avoiding high costs for equipment (such as liquid handling robots and arrays) and reducing those for reagents by two orders of magnitude will enable extensive research in synthetic biology, proteomics, medicine, and therapy, where the lack of well-characterized reagents limits progress.

## Supporting Information Available

Experimental details of cloning, vector construction, phage display panning, protein expression, and purification; flow cytometry procedures; documentation of screening outcomes; and kinetic and affinity analysis and practical procedures (PDF)
