# Supplementary material for: Multiplexed Affinity Characterization of Protein Binders Directly from a Crude Cell Lysate by Covalent Capture on Suspension Bead Arrays
Source: Anal Chem. 2021 Jan 5;93(4):2166–73. doi: 10.1021/acs.analchem.0c03992 (PMC7861142; doi:10.1021/acs.analchem.0c03992)
Supplement: Supplementary file 1 — ac0c03992_si_001.pdf [file ac0c03992_si_001.pdf]

# Supporting Information for

# Multiplexed affinity characterization of protein binders directly from crude cell lysate by covalent capture on suspension bead arrays

Tuomas Huovinen,[a] Laurens Lindenburg,[a] Ralph Minter[b,c] and Florian Hollfelder[a*]

[a] Department of Biochemistry, University of Cambridge, 80 Tennis Court Road, CB2 1GA, Cambridge, UK

[b] Antibody Discovery and Protein Engineering, R&D, AstraZeneca, Milstein Building, Granta Park, Cambridge, CB21 6GH, UK.

[c] Present address: Alchemab Therapeutics Ltd., 55-56 Russel Square, London, WC1B 4HP, UK.

* Corresponding author, email: fh111@cam.ac.uk

# Table of Contents


| | Page |
|---|---|
| 1. **Supplementary Materials and Methods** | S3 |
| 1.1 Construction of vectors | S3 |
| 1.2 Parental clones and construction of the site-directed mutagenesis library | S3 |
| 1.3 Phage display panning | S4 |
| 1.4 Protein expression | S5 |
| 1.5 Preparation of antigens and fluorescent conjugates | S5 |
| 1.6 Flow cytometry | |
| 1.7 Biolayer interferometry measurements | S6 |
| 2. **Supplementary Figures** | S7 |
| **Figure S1.** *Analysis of recombinant protein expression by SDS PAGE* | S7 |
| **Figure S2**. *Biotin-binding capacity of coated beads (Ø 5 µm) and the attachment of scFv-SpyCatcher* | S7 |
| **Figure S3.** *Bead sizes suitable for assembling multiplexed bead arrays.* | S8 |
| **Figure S4.** *Schematic overview of anti-DIG scFv phage library panning and SBA-based screening of the enriched clones as scFv-SpyCatcher clones* | S8 |
| **Figure S5.** *Bead gating of 24plex bead sample in the primary single concentration point screening of anti-DIG scFv-SpyCatcher clones covering wells 203A1-B12* | S9 |
| **Figure S6.** *Analysis of the equilibrium dissociation constant* $K_d$ *by multiplexed saturation binding bead assay* | S10 |
| **Figure S7.** *Kinetic analysis of scFv-SpyCatcher clones* | S10 |
| **3. Supplementary Tables** | S11 |
| **Table S1.** Primers used for site-directed mutagenesis of pET28a-T2H-SpyT and scFv 198C9 | S11 |
| **Table S2.** Digoxigenin-modified oligos for hybridization | S11 |
| **Table S3.** Screening summary of anti-digoxigenin scFv-SpyCatcher Clones | S12 |
| **Table S4.** Antigen (DIG-dsDNA-Cy5) saturation binding assay results for affinity ranking selected clones | S12 |
| **4. References** | S12 |

# 1. Supplementary Materials and Methods

### 1.1 Construction of vectors

Tamavidin-2-HOT gene was ordered in pET28a vector (Genscript) and a C-terminal SpyTag was added by site directed mutagenesis using primers SpyTins_Tama_F, SyTins_Tama_R, Tama-SpyT-STOP_ins_F and Tama_SpyT_STOP_ins_R (see Table S1 for sequences) creating a final expression construct pET28a-Tamavidin-2-HOT-SpyTag (referred to as T2H-SpyTag). The AA sequence of T2H-SpyTag was:

MGSSHHHHHHSSGLVPRGSHMSDVQSSLTGTWYNELNSKMELTANKDGTLTGKYLSKVGDVYVPYPLSGRYNLQPPAGQG
VALGWAVSWENSKIHSATTWSGQFFSESSPVILTQWLLSSSTARGDVWESTLVGCDSFTKTAPTEQQIAHAQLHCRAPRLKGS
AHIVMVDAYKPTK

The scFv-SpyCatcher expression vector pSC-scFv was constructed by replacing the C-terminal mouse CL domain gene in the periplasmic expression vector pLK01-scFv (see supplementary reference 1) with SpyCatcher gene (Genscript) using AarI sites. The core SpyCatcher sequence of 84 aa was used in all constructs[2]. The scFv-SpyCatcher expression vectors pET28a-scFv-SpyCatcher and pHBSC-scFv were created by cloning scFv-SpyCatcher as XbaI-HindIII fragment to pHB32x (modified from pEB32x (described in the supplementary reference 1) by adding a loxP element to the HindIII site for concatemer resolution by Cre recombinase in rolling circle amplification reactions) and pET28a (Novagen) vectors. The AA sequence for scFv-SpyCatcher clone 180B1 (with PelB signal sequence) was:

MKSLLPTAAAGLLLLAAQPAMAEIVLTQSPGTLSLSPGERATLSCRASQSVSSSSLHWYQQKPGQAPRLLIYGASSRATGVPDRF
SGSGSGTDFTLTISRLEPEDFAVYYCQQGNYAPHTFGQGTKVEIKRTGGGGSGAGGSGGGGTGGGGSEVQLLESGGGLVQPG
GSLRLSCAASGFTFSSYLMHWVRQAPGKGLEWVSSINPSGGGTYYADSVKGRFTISRDNSKNTLYLQMNSLRAEDTAVYYCVN
YLGYWGQGTLVTVSSASGAGSGAGGGSSGGGSGASAHLQSSDSATHIKFSKRDEDGKELAGATMELRDSSGKTISTWISDGQ
VKDFYLYPGKYTFVETAAPDGYEVATAITFTVNEQGQVTVNGHLQHHHHHH

### 1.2 Parental clones and construction of the site-directed mutagenesis library

The parental anti-digoxigenin scFv clones 180B1 and 198C9 were obtained by bead surface display selection (unpublished) from a human synthetic scFv library.[1] The sequence of 180B1 that was used for expression studies was (VH-linker-VL):

EIVLTQSPGTLSLSPGERATLSCRASQSVSSS**S**LHWYQQKPGQAPRLLIYGASSRATGVPDRFSGSGSGTDFTLTISRLEPEDFAVY
YC**Q**Q**GNYA**PHTFGQGTKVEIKRTGGGGSGAGGSGGGGTGGGGSEVQLLESGGGLVQPGGSLRLSCAASGFTFSSYLMHWV
RQAPGKGLEWVSSINPSGGGTYYADSVKGRFTISRDNSKNTLYLQMNSLRAEDTAVYYC**VNYLGY**WGQGTLVTVSS

The DNA sequence of scFv 180B1 gene (including the underlined SfiI sites used for cloning scFv repertoires between vectors) was:

ggcccagccggccatggcggaaattgtgctgacccaatctccgggcacactgagcttgtctccgggcgaacgtgcgacccttagctgcagagccagccagtcgg
tgtccagctcgtctcttcattggtatcaacagaaaccaggtcaagcacctcgcctgctgatttatggcgcctcttcacgtgccactggggtcccggatcgctttagc
ggctctggcagtggcaccgattttactctgaccatttcccgtctggaaccggaagacttcgcggtgtactattgtcagcaggggaactacgcccctcatacctttg
gccaggggacgaaagtcgagattaaacggacaggtggtggcggcagtggcgccggtgggtctggaggtggtggtaccggtggcggagggtcagaggtacag
ctgcttgaaagcggcggtggcctggtgcaaccgggtgggagcctgcgtctgtcgtgcgcagcctccggatttacgttctccagctacttgatgcattgggtccgtc
aggctccaggtaagggtctcgagtgggtcagttcgatcaatccttctggtgggggcacgtattatgcagatagcgtgaagggtcgcttcaccatctcccgggaca
attcgaagaacacactgtatctccaaatgaactcgctccgtgctgaggacactgccgtctactactgtgttaactacctgggttactggggtcagggtacactagt
caccgtgagctcggcctcgggggcc

The site-saturation libraries of CDR loops were constructed on clone 180B1 by SOE-PCR using Phusion DNA polymerase (Thermo). Residue positions in CDR-L1 (randomized positions are shown in bold and underlined: -SS**S**L**H**WY-; also annotated in the full sequence above), CDR-L3 (-YC**Q**Q**GNYA**P**H**TF-), CDR-H1 (not included in the final library), CDR-H2 (not included in the final library) and CDR-H3 (-YC**VNYLGY**WG-) were randomized using degenerate NNS codons. The primers used for mutagenesis are listed in Table S1. The scFv template gene was amplified with primer pairs TH147/TH157 (L1), TH147/TH161 (L3), TH147/TH162 (L3), TH147/TH164 (H2), TH147/TH168 (H3) and TH147/TH169 (H3) to generate the first half of the gene and the second half of the gene containing the substitutions was generated with primer pairs TH156/pIVBT7 (L1), TH158/pIVBT7 (L3), TH159/pIVBT7 (L3), TH160/pIVBT7 (L3), TH163/pIVBT7 (H2), TH165/pIVBT7 (H3), TH166/pIVBT7 (H3) and TH167/pIVBT7 (H3), respectively. The two parts had overlapping sequences that were joined together by SOE-PCR using TH147 and pIVBT7 primers reconstituting the full-length scFv sub-libraries. The final PCR products were pooled, cloned with SfiI sites into plasmid pCSpyT-scFv and transformed into E. cloni 10G cells (Lucigen) yielding $10^6$ cfu transformants.

The scFv clone 198C9 was identified later by bead surface display and, according to preliminary results (unpublished), showed higher binding signal for digoxigenin (DIG-dsDNA-fluorescein) than 180B1. Consequently, 198C9 was chosen as the parental clone for CDR site-saturation library construction and selection by phage display. Clone 198C9 differed from 180B1 by four substitutions of which E1K$_{VH}$ resides in the beginning of the VH domain and, V53A$_{VH}$, S55T$_{VH}$ and N57T$_{VH}$ in the CDR-H2 loop (underlined in the sequence above, numbering according to IMGT). Therefore, the site saturation library constructed on pCSpyT-scFv 180B1 was modified by replacing the region covering CDR-H1 and CDR-H2 diversity with a KpnI-SmaI fragment of 198C9 clone carrying all four substitutions. The final library repertoire was transferred as a SfiI cassette to phage display vector pHB32x. Transformation of pHB32x-scFv 198C9 library in SS320 cells yielded $10^6$ cfu transformants for phage display selections.

### 1.3 Phage display panning

Phage stock was prepared from scraped cells with VCS-M13 helper phage superinfection in 50 mL volume and precipitated twice with PEG/NaCl according to standard procedures.[1] $5x10^{12}$ phage particles were incubated in 1 mL PBS provided with 100 pM bio-dsDNA-DIG (dsLMB) overnight at 4 °C. The complexes were captured on paramagnetic MyOne streptavidin beads for 10 min, washed 3 x 1 mL PBST$^{0.1}$ and eluted with 100 µl elution buffer containing TBS with 100 µg/mL trypsin, 0.05 U/µl benzonase (Millipore) and 2.5 mM $MgCl_2$ for 15 min at RT with shaking. The beads were collected on magnet and the supernatants were infected to mid-log SS320 cells (Lucigen). After 30 min incubation at 37 °C the cells were plated on LA supplemented with 25 µg/mL chloramphenicol, 10 µg/mL tetracycline and 0.5% w/v glucose and incubated o/n at 37 °C.

New phage stock was prepared from scraped cells in 10 mL cell culture volume as above. The second round of panning was performed with $5x10^9$ phage particles in the presence of 20 pM and 1 pM bio-dsDNA-DIG in 2 mL PBST$^{0.05}$ for 15 min at RT followed by capture on streptavidin beads, washing, elution and infection as above. From the no-antigen control, 1 pM antigen and 20 pM antigen selection, a total of 500, 1800 and 58 000 cfu were obtained, respectively.

### 1.4 Protein expression

*E. coli* strain BL21(DE3) was used for the expression of proteins for NiNTA purification. T2H-SpyTag was expressed using vector pET28a-Tamavidin-2-HOT-SpyTag. ScFv-SpyCatcher clone 180B1 was expressed with three different vectors for expression comparison, pSC (ampicillin resistant), pHBSC (chloramphenicol resistant) and pET28a (kanamycin resistant) (see Figure S1), but later only the pHBSC-vector was used for further studies. For small scale experiments T2H protein fusion was expressed in 10 mL LB and scFv-SpyCatchers in 50 mL SB. The cultures were grown at 37 °C until OD 0.6, induced with 200 μM Isopropyl-β-D-thiogalactopyranoside (IPTG) and incubated overnight at 26 °C. Pelleted cells were lysed in 1 mL BugBuster (1x; Novagen, EMD Chemicals Inc., San Diego, CA) in PBS, supplemented with 25 U/mL benzonase (Novagen). After pelleting the cell debris, the supernatant was purified using protein miniprep spin columns (Zymo Research Corp, Orange, CA) according to manufacturer´s instructions. Large scale expression and lysis was carried out as above, but scaled up to a 1 L volume. 3 mL NiNTA matrix was used for the purification of tamavidin-SpyTag expression and 0.5 mL for SpyCatcher-scFv expressions. The supernatant was loaded onto the column in the presence of 5 mM imidazole, washed with 20 mM imidazole in PBS and eluted with 500 mM imidazole in PBS. The eluted proteins from the large-scale expressions were concentrated and the buffer was exchanged to PBS (3x) by ultracentrifugation using Amicon Ultra-4 centrifugal filter devices (10,000 MWCO, EMD Millipore Corporation, Billerica, MA).

### 1.5 Preparation of antigens and fluorescent conjugates

DIG-dsDNA-bio and DIG-dsDNA-Cy5 for phage panning and flow cytometry screening were prepared by hybridizing oligonucleotides (Sigma) listed in Table S2. Oligos were dissolved in $H_2O$ at 100 μM stock concentration and mixed with annealing buffer (AB, 100 mM Potassium Acetate; 30 mM HEPES, pH 7.5) in volume ratios of 1:1:2 (TH135:TH212:AB) for biotinylated digoxigenin and 1:1:2 (TH210:TH211:AB) for Cy5-labeled digoxigenin, providing a final 25 μM concentration for the duplex. To hybridize the oligos, the reactions were heated to 94°C for 2 minutes and transferred from the heating block to the bench for slow cooling to room temperature. The DIG-DNA conjugates were stored frozen for later use.

*Bio-BSA-ATTO-565.* BSA (Sigma) was dissolved in 1 mL PBS to 1 mmol/l (1 μmol) concentration and supplemented with 158 μl 20 mM (3.2 μmol) Biotin-amidohexanoyl-6-aminohexanoic acid N-hydroxysuccinimide ester (Sigma) dissolved in DMSO. The reaction was incubated o/n at RT on rotation, quenched with 300 μl 1 M Tris-Cl pH 8 for 10 min and desalted with a PD10 (GE) column equilibrated with PBS. The biotinylated BSA was concentrated with Amicon Ultra 10,000 MWCO ultrafiltration device (EMD Millipore) to 1 mL PBS and reduced with 20 x molar excess of tris(2-carboxyethyl)phosphine (TCEP, 20 μmol) in 1 mL PBS at RT for 30 min. 1 mg of ATTO-565 maleimide (ATTO-TEC) was dissolved in 50 μl DMF and added step-wise to reduced BSA. The labelling reaction was incubated on rotation at RT for 5 h protected from light. The reaction was quenched with 10 mM DTT for 15 min at RT and desalted by a PD10 column equilibrated with PBS. The elution from the PD10 column was collected in three fractions and the fraction with 82 μM concentration was used for bead coating (degree of labelling ATTO-565/BSA: 0.8).

### 1.6 Flow cytometry.

A BD FACScan (BD Biosciences, San Jose, CA) updated with DxP8 package (Cytek Biosciences, Fremont, CA) was used for collecting flow data. The instrument contained three

lasers with excitation maxima at 488 nm (blue), 561 nm (yellow-green) and 633 nm (red). FITC fluorescence was followed on BluFL1 (emission band pass filter set (BP): 530/30 nm), ATTO-565 on YelFL2 (BP: 615/25 nm) and Cy5 on RedFL1 channel (BP: 666/27 nm). Data were analyzed with FlowJo10 software (FlowJo, LLC, Ashland, OR). The files were first processed by gating the events by bead size (FSC/SSC-plot) followed by gating individual address bead populations based on their BluFL1/YelFL2-fluorescence ratio (as shown in Figure 3). Functional signal, i.e., the fluorescence of the Cy5-labeled antigen bound to the beads, was measured as the median fluorescence intensity of individual bead populations in the RedFL1 channel.

### 1.7 Biolayer interferometry measurements

$K_d$ values for scFv-SpyCatcher 198C9 and 203A2 were determined using an Octet Red96 instrument (ForteBio, Inc.) in $PBST^{0.002}$ at 30 °C with streptavidin (SA)-coated sensor tips (ForteBio, Inc.) shaking at 1000 rpm. Biotin-DIG was used as the immobilized ligand and loaded on the tip at 25 nM concentration for 300 s. Purified scFv-SpyCatcher proteins were used as the analyte allowing 300 s for association and 600 s for dissociation phase, respectively. The molar concentration of the purified scFv-SpyCatcher proteins was determined by measuring the absorbance at 280 nm and using an extinction coefficient of 50 690 $M^{-1}cm^{-1}$ calculated with Vector NTI software 5.5 (ThermoFisher Scientific) from the sequence. The binding of the clone 198C9 was followed in a two-fold dilution series starting at 100 nM concentration. Clone 203A2 was measured similarly starting at 50 nM concentration. The data were analyzed using the Octet Software (ForteBio Inc.). The signal obtained from a reference sensor without the analyte was subtracted from the sample traces and the binding constants were determined using a 1:1 Langmuir-binding model in the kinetic analysis.

## 2. Supplementary Figures

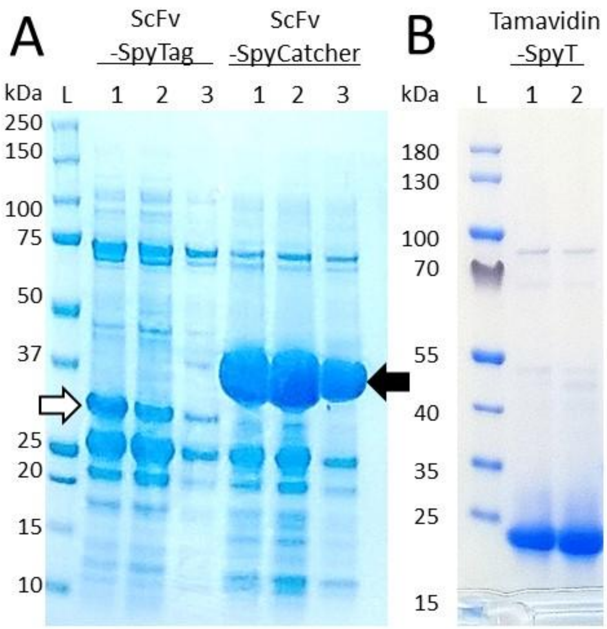


**Figure S1.** *Analysis of recombinant protein expression by SDS PAGE.* A) Samples of spin column NiNTA purification of anti-DIG clone 180B1 as scFv-SpyTag (30.2 kDa, white arrow) and scFv-SpyCatcher (38.2 kDa, black arrow) fusions expressed in 50 mL cell culture with vectors pSC (1), pHBSC (2) and pET28a (3). B) Samples of spin column NiNTA purification of Tamavidin-SpyTag (19.2 kDa) expressed in10 mL cell culture with pET28a in two replicates.

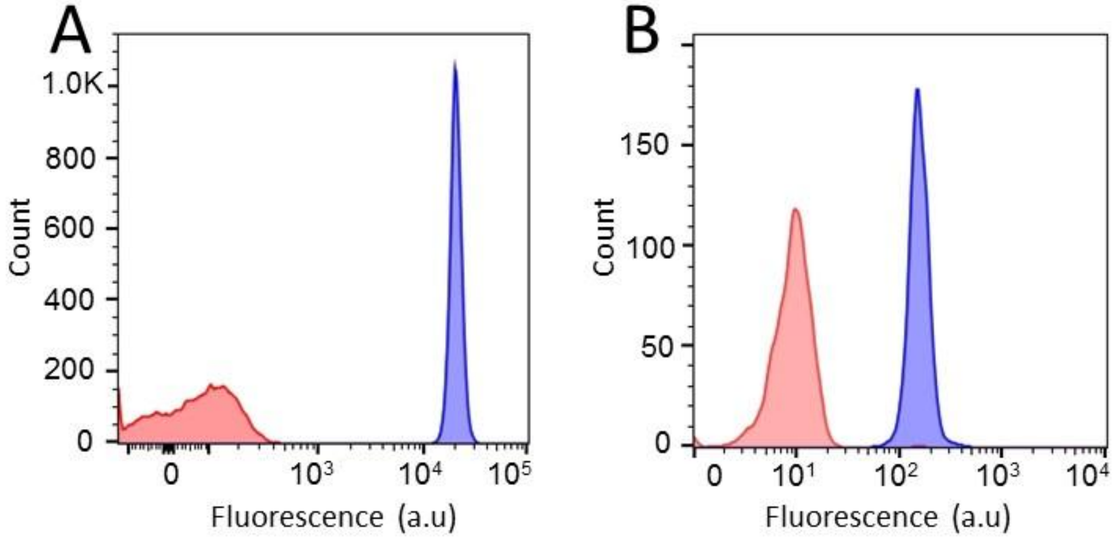


**Figure S2**. *Biotin-binding capacity of coated beads (Ø 5 µm) and the attachment of scFv-SpyCatcher.* A) Biotin binding was retained after covalent attachment of tamavidin-SpyTag on the beads. Flow cytometric analysis of $10^5$ beads coated with (blue) and without (red) tamavidin-SpyTag and labeled with 5 µM biotinylated fluorescein. B) Flow cytometric analysis of $10^4$ tamavidin-SpyTag beads coated with (blue) and without (red) scFv-SpyCatcher clone 180B1 and subsequently labeled with DIG-dsDNA-Cy5 (25 nM).

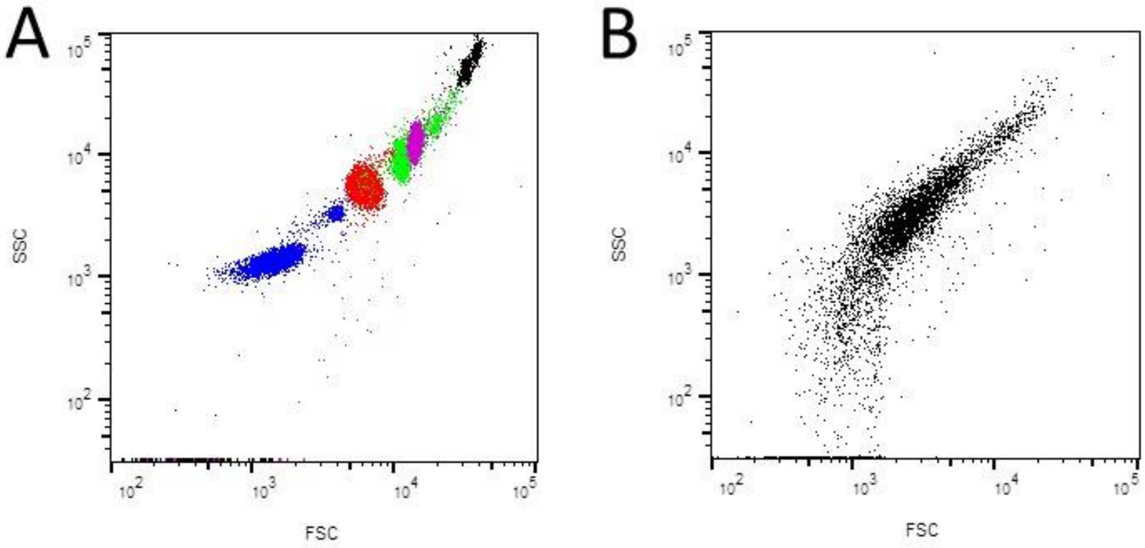


**Figure S3.** *Bead sizes suitable for assembling multiplexed bead arrays.* A) Scatter plot of paramagnetic carboxylic acid beads with the mean diameter of 2.8 µm (blue), 5.2 µm (red), 7.4 µm (green), 10.3 µm (purple) and 18.8 µm (black) run separately and overlaid for visualization. B) Scatter plot of Ø 1.43 µm bead size. The smallest beads (Ø 1.43 µm) show wide scatter profile and Ø 7.4 µm beads overlap with Ø 10 µm bead size. As the bead count of the Ø 19 µm stock was at least five-fold lower than in the other stocks, only Ø 5 and 10 µm beads were employed in subsequent experiments.

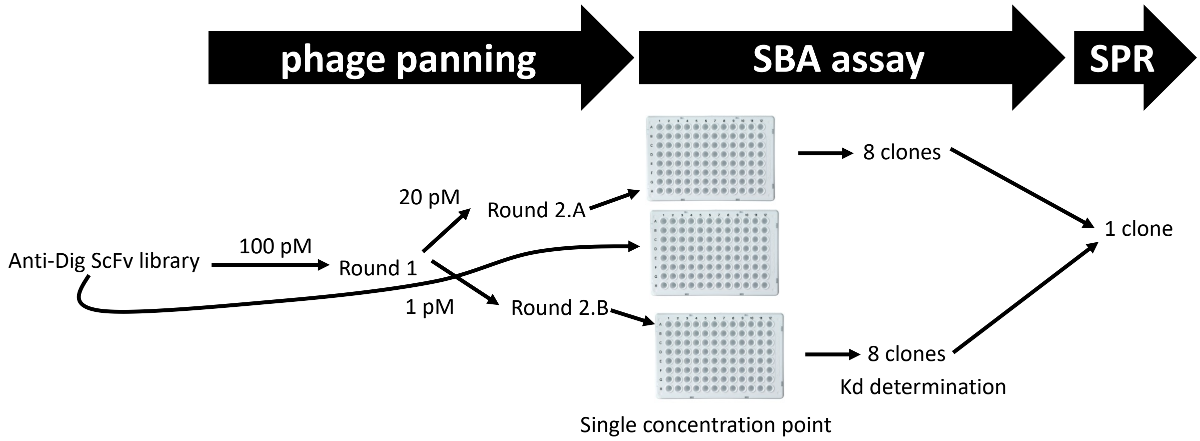


**Figure S4.** *Schematic overview of anti-DIG scFv phage library panning and SBA-based screening of the enriched clones as scFv-SpyCatcher clones.* After primary screening, eight top scFv clones (by S/B ratio) were ranked with a saturation binding assay for affinity and the affinity of the best scFv clone 203A2 was determined by biolayer interferometry.

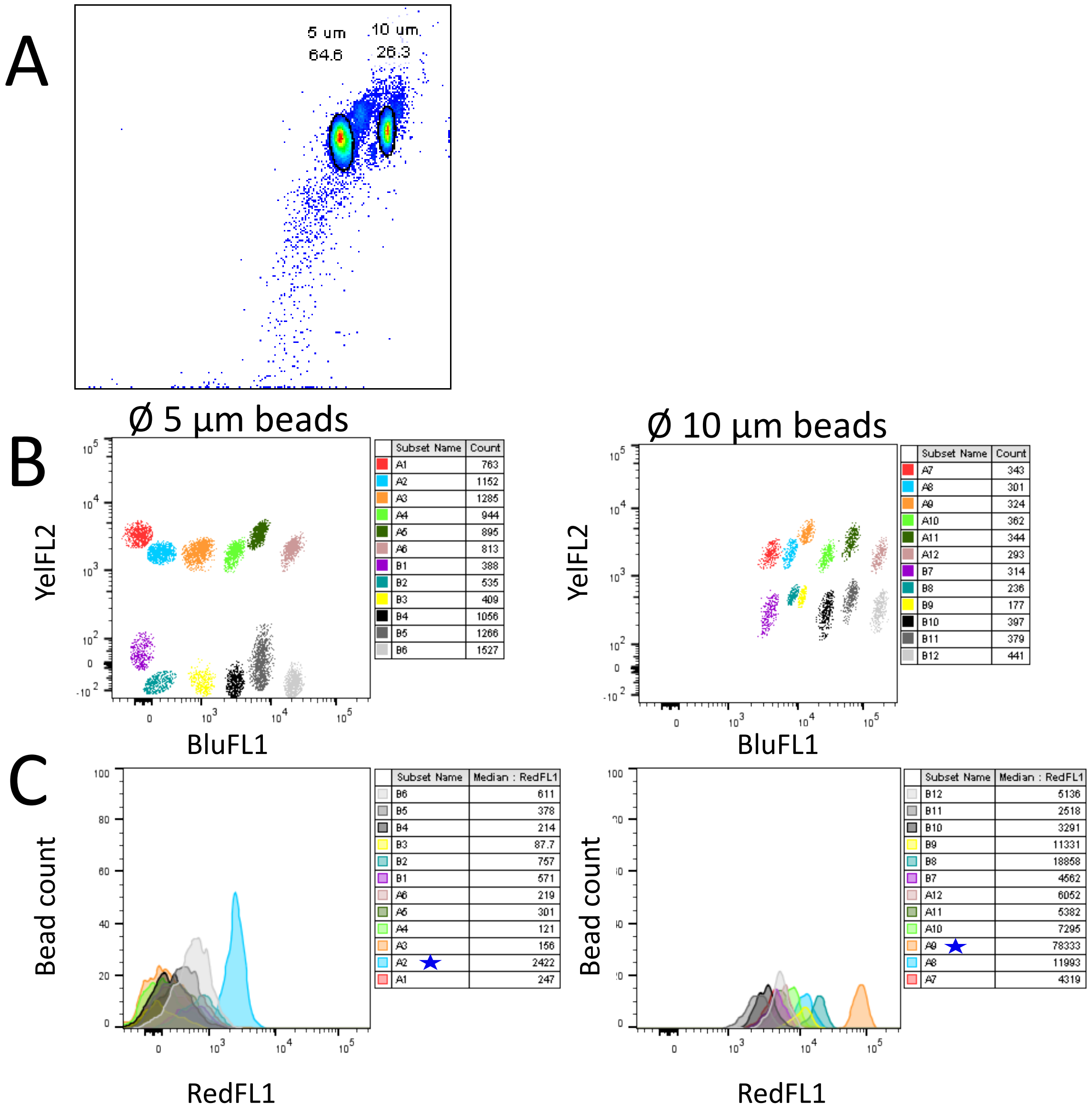


**Figure S5.** *Bead gating of the 24-plex bead sample in the primary single concentration point screening of anti-DIG scFv-SpyCatcher clones covering wells 203A1-B12.* A) FSC/SSC-plot of color-coded beads with gates resolving beads of ø 5 µm (64.6 % of all events) and ø 10 µm (26.3% of all events) into subgroups. Both bead populations contain a small amount of doublets visible next to the main population. B) Beads were further gated into subpopulations based on BluFL1/YelFL2-plot and annotated by their well coordinates. Rows 1-6 contain ø 5 µm beads and rows 7-12 ø 10 µm beads, respectively. C) Median fluorescence intensity obtained with 5 nM digoxigenin-dsDNA-Cy5 label incubation for ranking the clones by their antigen binding affinity. Clone 203A2 found from the ø 5 µm bead screening (blue star) was later identified to have the highest affinity for the antigen. Clone 203A9 originating from the ø 10 µm bead screening (blue star) was later identified to be genetically identical to clone 204C6.

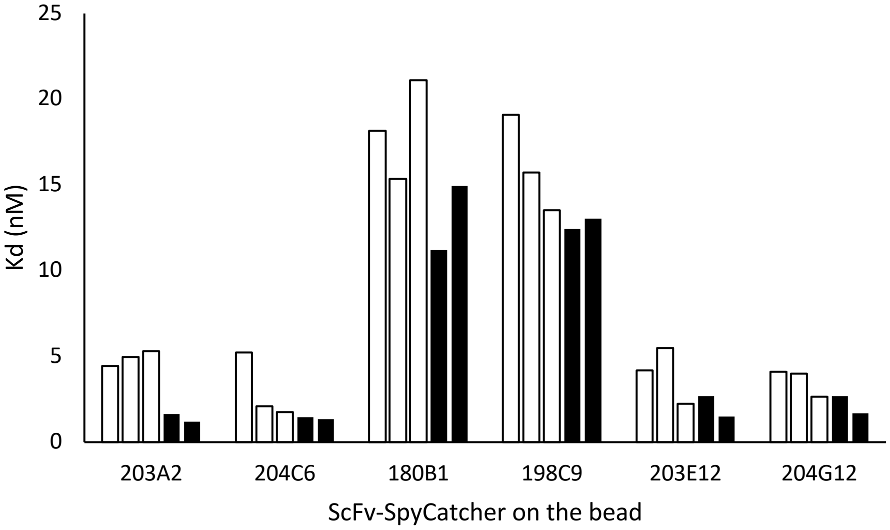


**Figure S6.** *Analysis of the equilibrium dissociation constant $K_d$ by multiplexed saturation binding bead assay.* The equilibrium dissociation constants were determined by saturation plot analysis in a series of antigen dilutions (600 nM – 16 pM DIG-dsDNA-Cy5) provided in 200 µl (white) and 2 mL (black) volumes. The individual $K_d$ values were measured by attaching the clone in multiple experiments to a different Ø 5 µm color address bead batch containing FITC (intensities: 1, 3, 4, 5 and 6) and bio-ATTO-565 (intensities: ++ and -) as indicated in Figure 3B.

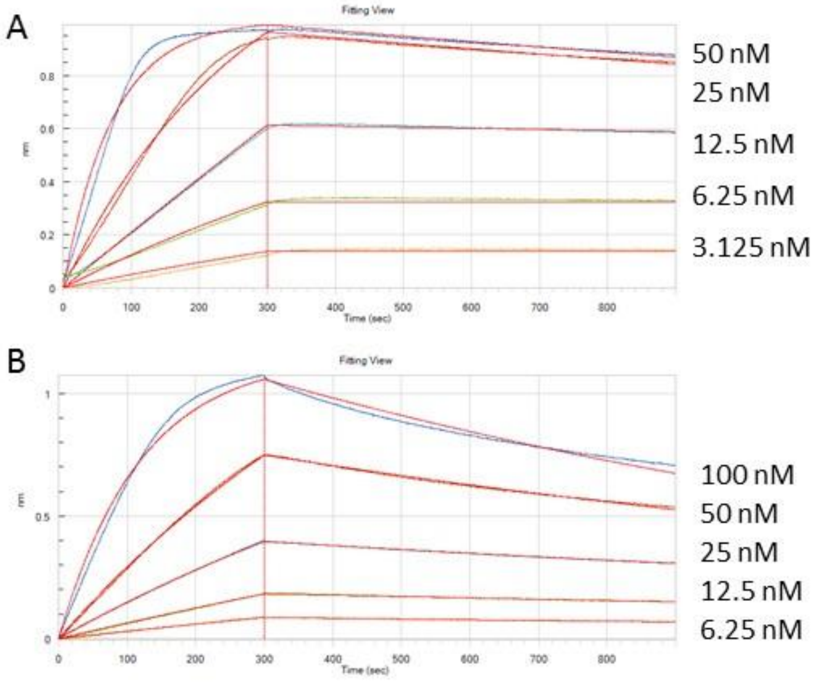


**Figure S7.** *Kinetic analysis of scFv-SpyCatcher clones.* (A) 203A2 ($K_d$: 1.2 ± 0.6 nM) and (B) the parental clone 198C9 ($K_d$: 21.4 ± 9.8 nM) with biolayer interferometry. Bio-dsDNA-DIG was immobilized on streptavidin tips and the binders were added as analyte at five different concentrations to record the binding curves.

# 3. Supplementary Tables

**Table S1.** Primers used for site-directed mutagenesis of pET28a-T2H-SpyT and scFv 198C9.

| Oligo name | Sequence (5′-3′) |
|---|---|
| SPYTINS_TAMA_F | GTGGACGCCTACAAGCCGACGAAGGCTAACAAAGCCCGAAAGGAAGCTG |
| SPYTINS_TAMA_R | GGCTTGTAGGCGTCCACCATCACGATGTGGCAGAGCCTTTCAGACGCGGTGCACG |
| TAMA_SPYT_STOP_INS_F | CCGACGAAGTAAGCTAACAAAGCCCGAAAGGAAGCTG |
| TAMA_SPYT_STOP_INS_R | CTTTGTTAGCTTACTTCGTCGGCTTGTAGGCGTC |
| TH147 | CTCACTATAGGGAGACCACAACGGTTTCCC |
| pIVBT7 | AGGGGTTATGCTAGTTATTGCTCAGCGGTG |
| TH156 L1F | GCCAGCCAGTCGGTGTCCAGCNNSNNSCTTNNSTGGTATCAACAGAAACCAGGTCAAG |
| TH157 L1R | GCTGGACACCGACTGGCT |
| TH158 L3F1 | GACTTCGCGGTGTACTATTGTNNSCAGNNSAACTACGCCCCTCATACCTTTGGCC |
| TH159 L3F2 | GTGTACTATTGTCAGCAGGGGNNSNNSGCCCCTCATACCTTTGGCCAGG |
| TH160 L3F3 | GTGTACTATTGTCAGCAGGGGAACTACNNSCCTNNSACCTTTGGCCAGGGGACGAAAGTC |
| TH161 L3R1 | ACAATAGTACACCGCGAAGTC |
| TH162 L3R2 | CCCCTGCTGACAATAGTACAC |
| TH163 H2F | AAGGGTCTCGAGTGGGTCAGTNNSATCNNSCCTTCTGGTGGGGGCACGTATTATG |
| TH164 H2R | ACTGACCCACTCGAGACCCTT |
| TH165 H3F1 | CACTGCCGTCTACTACTGTNNSNNSTACCTGGGTTACTGGGGTCAGG |
| TH166 H3F2 | CACTGCCGTCTACTACTGTGTTAACNNSNNSGGTTACTGGGGTCAGGGTACAC |
| TH167 H3F3 | CGTCTACTACTGTGTTAACTACCTGNNSNNSTGGGGTCAGGGTACACTAGTCAC |
| TH168 H3R1 | ACAGTAGTAGACGGCAGTG |
| TH169 H3R2 | CAGGTAGTTAACACAGTAGTAGACG |

**Table S2.** Digoxigenin-modified oligos for hybridization.

| Oligo name | Sequence (5′-3′) |
|---|---|
| TH135_DIG-LMB | [digoxigenin]-ATGTGCTGCAAGGCGATTAAG |
| TH212 biotin-anti-LMB | [biotin]-CTTAATCGCCTTGCAGCACAT |
| TH210 pIV-DIG | [digoxigenin]-GCGTTGATGCAATTTCTATGC |
| TH211 Cy5-pIVrev | [Cy5]-GCATAGAAATTGCATCAACGC |

**Table S3.** Screening summary of anti-digoxigenin scFv-SpyCatcher clones.

| Plate | Source[a] | Multiplexing | Antigen[b] | Positive/ Screened[c] | Unique/ Sequenced[d] |
|---|---|---|---|---|---|
| 201 | Input library | 12plex | 1 mL 5 nM | 42/84 | N.D. |
| 204 | 2nd Round output A | 12plex (24plex[e]) | 0.25 mL 5 nM | 72/84 (64/84) | 2/4 |
| 203 | 2nd Round output B | 24plex[e] | 0.25 mL 5 nM | 69/84 | 2/5 |

[a] The second round of phage display was performed with 5x $10^9$ infective phage particles by incubating with bio-dsDNA-DIG in 1 mL $PBST^{0.05}$ at 20 pM (output A) and 1 pM (output B) antigen concentration for 30 min followed by capture on paramagnetic streptavidin beads.
[b] The antigen DIG-dsDNA-Cy5 was provided in $PBST^{0.05}$ and incubated for 1h at RT on rotation.
[c] Cut-off for 5 nM label incubation: 3x S/B.
[d] 8 clones were re-screened from each primary plate in a secondary screening assay using saturation binding (201, 204, 203). The highest-ranking clones in the secondary screening assay were sequenced and unique clones taken forward for repeated affinity determination.
[e] The two lowest intensity ATTO-565 bead arrays (+ and –) were used for multiplexing.

**Table S4.** Antigen (DIG-dsDNA-Cy5) saturation binding analysis for affinity ranking of selected clones.

| Input library (plate 201) | $K_d$[a] [nM] | $R^2$ | Output library A (plate 204) | $K_d$[a] [nM] | $R^2$ | Output library B (plate 203) | $K_d$[a] [nM] | $R^2$ |
|---|---|---|---|---|---|---|---|---|
| 201C9 | 23.7 | 0.95 | 204E11 | 0.9 | 0.98 | 203A2 | 1.7 | 0.97 |
| 201E9 | 25.6 | 0.96 | 204C6 | 1.5 | 0.99 | 203E12 | 4.3 | 0.96 |
| 201E2 | 26 | 0.98 | 204F12 | 2.8 | 0.97 | 203D1 | 8.6 | 0.96 |
| 201G8 | 26.2 | 0.95 | 204G12 | 3.6 | 0.98 | 203E8 | 9.3 | 0.95 |
| 201G9 | 37.2 | 0.96 | 204E5 | 4.9 | 1 | 203E2 | 12.1 | 0.97 |
| 201C3 | 69.1 | 0.96 | 204E6 | 5.2 | 1 | 203G8 | 18.7 | 0.95 |
| 201F3 | 79.8 | 0.97 | 204F8 | 14.4 | 0.95 | 203F1 | N.D. | <0.95 |
| 201C1 | N.D. | <0.95 | 204C2 | N.D. | <0.95 | 203A9 | N.D. | <0.95 |

[a] $K_d$ values were determined with 12-plex SBAs from single concentration gradient of DIG-dsDNA-Cy5.

# 4. References


(1) Huovinen T., Syrjänpää M., Sanmark H., Brockmann E.C., Azhayev A., Wang Q., Vehniäinen M. and Lamminmäki U. (2013) Two ScFv antibody libraries derived from identical VL-VH framework with different binding site designs display distinct binding profiles. *Protein Eng Des Sel.* **26**(10), 683-693, doi: 10.1093/protein/gzt037.

(2) Li L., Fierer J.O., Rapoport T.A. and Howarth M. (2014) Structural analysis and optimization of the covalent association between SpyCatcher and a peptide Tag. *J Mol Biol,* **426**, 309-317, doi: 10.1016/j.jmb.2013.10.021.
